# Parks and Recreation: A Social Driver of Healthy Aging

**DOI:** 10.1093/geroni/igaf122.1576

**Published:** 2025-12-31

**Authors:** Dianne Palladino, Austin Barrett, Natalia Ospina, Colleen Pittard, Allison Colman, Melissa May, Eydie Kramer-Kostecka

**Affiliations:** National Recreation and Park Association, Ashburn, Virginia, United States; National Recreation and Park Association, Ashburn, Virginia, United States; National Recreation and Park Association, Ashburn, Virginia, United States; National Recreation and Park Association, Ashburn, Virginia, United States; National Recreation and Park Association, Ashburn, Virginia, United States; National Recreation and Park Association, Ashburn, Virginia, United States; National Recreation and Park Association, Ashburn, Virginia, United States

## Abstract

According to NRPA’s Supporting Older Adults Through Parks and Recreation report, 92% of park and recreation (P&R) agencies offer resources and programs for older adults, and 95% consider improving social connection and reducing social isolation the top benefits of older adult programming. However, 30% of agencies currently offer intergenerational programming, identifying an opportunity for P&R agencies to fill a gap in this need for supporting older adults’ physical and mental health. With support from RRF Foundation for Aging, NRPA responded to the need for more research on community-based solutions through P&R that increase intergenerational connectedness. Along with the survey of park and recreation agencies, focus groups of interdisciplinary experts in the healthy aging space revealed seven key pathways to advance health equity and social and intergenerational connectedness through P&R (centering equity, stakeholder engagement, equitable partnerships, co-location of services/spaces, empowerment/reciprocity, key messaging/mindsets, diversity of programs). We also conducted structured interviews with five diverse P&R agencies currently offering programs focused on encouraging connection between older adults and youth, resulting in case studies and summaries that highlight implementation, successes, challenges, best practices, and lessons learned. Through these findings, we created the Healthy Aging Framework, A Guide to Supporting Healthy Aging Through P&R. With continuing support from RRF, NRPA is conducting a follow-up intervention study to test the framework with four diverse P&R agencies that wish to establish sustainable programming to promote intergenerational connectedness and physical and mental health in older adults in the communities in which they live, learn, work, play, and age.

